# Polyporusterone E, a Key Component of *Polyporus umbellatus*, as a Potential Regulator of CHEK 1 in Liver Cancer: Integrated Network Pharmacology, Bioinformatics, and Experimental Validation

**DOI:** 10.3390/ijms27135694

**Published:** 2026-06-24

**Authors:** Xinhui Huang, Ruichen Gao, Xinran Yu, Zheng Feng, Qingxia Wang, Xiaotian Wu, Shulu Zhang, Yinze Zhong, Yeqing Xu, Meiting Jiang, Chunli Gan, Xiaotong Wang, Shuang Jiang, Chunjuan Yang

**Affiliations:** 1 Department of Pharmaceutical Analysis and Analytical Chemistry, College of Pharmacy, Harbin Medical University, Harbin 150088, China; 2 Department of Medicinal Chemistry and Natural Medicine Chemistry, College of Pharmacy, Harbin Medical University, Harbin 150088, China

**Keywords:** hepatocellular carcinoma, *Polyporus umbellatus*, polyporusterone E, CHEK1, network pharmacology

## Abstract

Hepatocellular carcinoma (HCC) is a lethal malignancy requiring novel therapeutic interventions. While *Polyporus umbellatus* exhibits anti-tumor properties, its specific bioactive pharmacophores and molecular mechanisms remain elusive. This study integrated network pharmacology, computational simulation, and experimental validation to decipher the anti-HCC efficacy of *Polyporus umbellatus*. Screening identified 11 bioactive sterols, with intersection analysis revealing 63 core targets. Clinical data stratified Checkpoint Kinase 1 (CHEK1) as a critical high-risk oncogene associated with poor prognosis. Molecular dynamics simulations (100 ns) demonstrated that polyporusterone E, a key constituent, forms a thermodynamically stable complex with CHEK1 via high-affinity hydrogen bonding. In vitro assays in HepG2 and HuH-7 cells confirmed that CHEK1 overexpression drives proliferation and metastasis, while its silencing reverses these phenotypes. Crucially, treatment with *Polyporus umbellatus* extract and purified polyporusterone E significantly compromised HCC cell viability and downregulated CHEK1 expression at transcriptional and translational levels. These findings suggest that polyporusterone E may downregulate CHEK1 expression and modulate CHEK1-associated signaling in HCC cells, providing preliminary evidence for the molecular basis of *Polyporus umbellatus* and highlighting its potential as a complementary therapeutic strategy for HCC management.

## 1. Introduction

Hepatocellular carcinoma (HCC) represents a significant global health burden, ranking as the sixth most common malignancy and the third leading cause of cancer-related mortality worldwide [1,2]. The incidence of HCC is projected to rise substantially, potentially reaching 1.4 million diagnoses by 2040, driven by shifting etiologies from viral hepatitis to non-viral factors such as metabolic dysfunction-associated steatotic liver disease (MASLD) and alcohol-related liver disease (ALD) [3,4]. This epidemiological transition underscores the urgent need for innovative therapeutic strategies, as current treatments, including surgical resection, transplantation, and systemic therapies such as immune checkpoint inhibitors, face limitations, including drug resistance, tumor heterogeneity, and high recurrence rates [5]. Traditional Chinese Medicine (TCM), with its multi-component and multi-target approach, has emerged as a complementary modality that offers potential benefits in enhancing anti-tumor immunity, reducing side effects, and improving patient survival [6,7].

Recent advancements in TCM research have highlighted the efficacy of herbal formulations in HCC management, often elucidated through network pharmacology, which integrates bioinformatics to predict active compounds, targets, and pathways [8,9,10]. Indeed, emerging evidence indicates that complex signaling cascades, such as transcription factor-mediated regulatory loops, play pivotal roles in driving cancer progression across multiple malignancies [11]. For instance, herbs such as Scutellariae Radix and Shuanglian Decoction have demonstrated anti-HCC effects by modulating pathways such as PI3K-Akt and MAPK, inhibiting proliferation, and inducing apoptosis [12]. *Polyporus umbellatus*, a medicinal fungus used in TCM for diuretic and anti-tumor effects, contains bioactive polysaccharides and sterols that exhibit immunomodulatory, antioxidant, and cytotoxic activities against various cancers, including liver tumors [13]. Key compounds such as polyporusterone E have shown promise in suppressing tumor growth, though primarily in breast and lung cancer models [14,15]. Additionally, checkpoint kinase 1 (CHEK1), a serine/threonine kinase involved in the DNA damage response and cell cycle regulation, is overexpressed in HCC and correlates with advanced stages, poor prognosis, and enhanced proliferation and invasion [16,17]. Despite these insights, significant gaps persist in understanding the precise molecular mechanisms of *Polyporus umbellatus* in HCC, particularly how its bioactives interact with key oncogenic targets such as CHEK1. Existing studies on TCM for HCC often lack an integrated approach that combines network pharmacology with experimental validation, leaving uncertainties regarding synergistic effects and clinical translation.

The present study employs a network pharmacology approach to screen bioactive compounds from *Polyporus umbellatus*, predict targets using tools such as SwissTargetPrediction and STRING, and identify core intersections with HCC-related genes from databases such as GeneCards and TCGA-LIHC. We focus on polyporusterone E’s interaction with CHEK1, validated through molecular docking, dynamics simulations, and in vitro assays in HepG2 and HuH-7 cells, demonstrating suppression of proliferation, migration, and invasion via CHEK1 downregulation. Enrichment analyses reveal involvement in pathways such as PI3K-Akt and the cell cycle, and prognostic analyses confirm CHEK1’s role in poor outcomes.

This research elucidates the multi-target mechanisms of *Polyporus umbellatus* in HCC, positioning polyporusterone E as a novel potential CHEK1 modulator with therapeutic potential. By integrating computational predictions with empirical validation, this approach advances TCM modernization, offering insights into developing adjunctive therapies to improve HCC outcomes and address resistance to conventional treatments.

## 2. Results

### 2.1. Pharmacological Profiling and Compound–Target Network Architecture of Polyporus umbellatus

**Figure 1:**
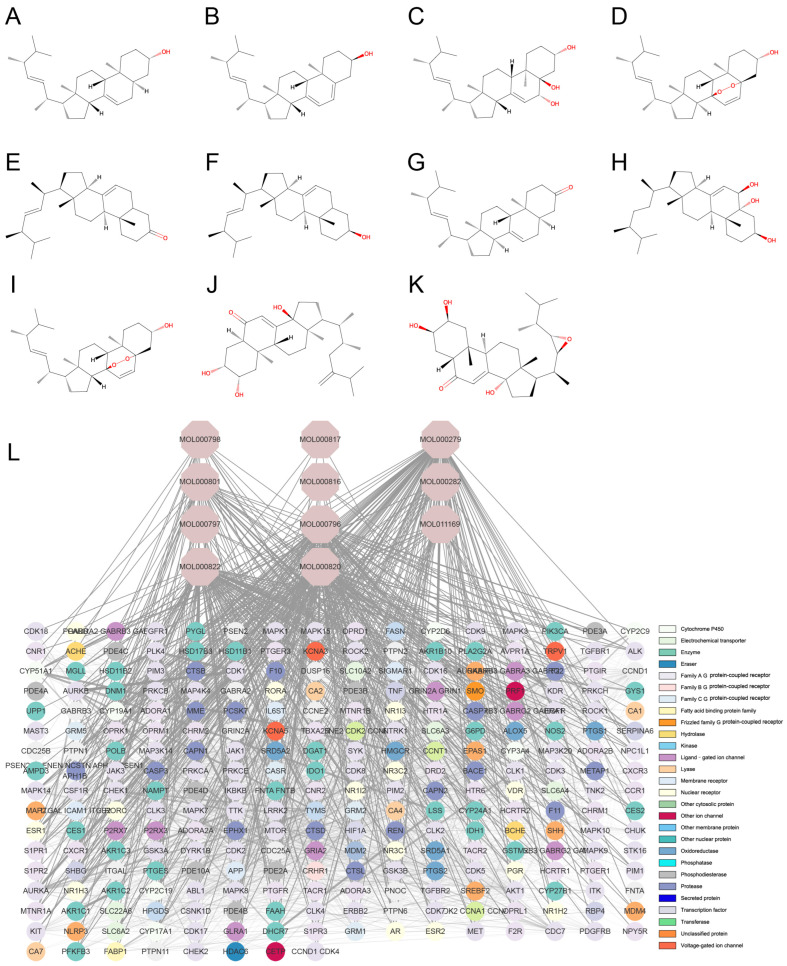
Chemical structures of the active compounds in *Polyporus umbellatus* and the compound–target network. (**A**) Chemical structure of ergosta-7,22-diene-3β-ol (MOL000798). (**B**) Chemical structure of ergosta-5,7,22-trien-3-ol (MOL000817). (**C**) Chemical structure of Cerevisterol (MOL000279). (**D**) Chemical structure of 5alpha,8alpha-epidioxy-(22e,24r)-ergosta-6,22-dien-3beta-ol (MOL000801). (**E**) Chemical structure of ergosta-7,22-dien-3-one (MOL000816). (**F**) Chemical structure of ergosta-7,22E-dien-3beta-ol (MOL000282). (**G**) Chemical structure of (22e,24r)-ergosta-7,22-dien-3-one (MOL000797). (**H**) Chemical structure of (22e,24r)-ergosta-6-en-3beta,5alpha,6beta-triol (MOL000796). (**I**) Chemical structure of Peroxyergosterol (MOL011169). (**J**) Chemical structure of polyporusterone G (MOL000822). (**K**) Chemical structure of polyporusterone E (MOL000820). (**L**) The compound–target (C-T) network of *Polyporus umbellatus*. The pink hexagonal nodes represent the 11 active candidate compounds corresponding to structures (**A**–**K**). The colored circular nodes represent the potential protein targets. Edges indicate the interactions between the compounds and their targets. The colors of the target nodes denote different protein functional classes, as indicated in the legend on the right.

The investigation into the therapeutic mechanisms of *Polyporus umbellatus* commenced with a comprehensive characterization of its material basis, revealing a constellation of bioactive steroidal compounds that likely underpin its anti-tumor efficacy. Using ADME-based screening protocols, we identified 11 core active ingredients with favorable pharmacokinetic properties. These compounds are predominantly ergosterol derivatives, which are structurally significant due to their established roles in modulating cellular signaling and immune responses. As illustrated in the structural panels, the identified agents include ergosta-7,22-diene-3β-ol, ergosta-5,7,22-trien-3-ol, and Cerevisterol, among others, such as polyporusterone G and E (Figure 1A–K). The structural diversity of these ergostane-type triterpenoids suggests a broad spectrum of biological activities, potentially enabling the herbal formulation to intervene in pathological processes through distinct yet complementary chemical pathways [18,19].

To further decipher the molecular interplay between these constituents and the host biological system, we constructed a compound–target network (Figure 1L). This bipartite graph provides a panoramic view of the pharmacological interactome. The network topology reveals a dense connectivity pattern in which the 11 active compounds (pink hexagonal nodes) interact with numerous protein targets (colored circular nodes). This visualization underscores the “multi-component, multi-target” therapeutic paradigm characteristic of traditional medicine. Notably, the network demonstrates that single compounds, such as ergosta-7,22-diene-3β-ol, can modulate multiple distinct targets, a phenomenon known as polypharmacology. Conversely, specific key targets are regulated by multiple compounds simultaneously, suggesting a synergistic effect where the combined action of these ingredients may produce a therapeutic outcome superior to that of individual agents. The target nodes are color-coded by protein functional classes, revealing that *Polyporus umbellatus* likely exerts its effects by influencing a wide array of biological functions, including enzyme activity, membrane receptor signaling, and transcriptional regulation. This intricate connectivity implies that the anti-hepatocarcinoma effects of *Polyporus umbellatus* are not attributable to a single “magic bullet” but rather to a holistic modulation of the cancer-associated protein network.

### 2.2. Identification of Critical Therapeutic Targets and Prognostic Stratification in Hepatocellular Carcinoma

**Figure 2:**
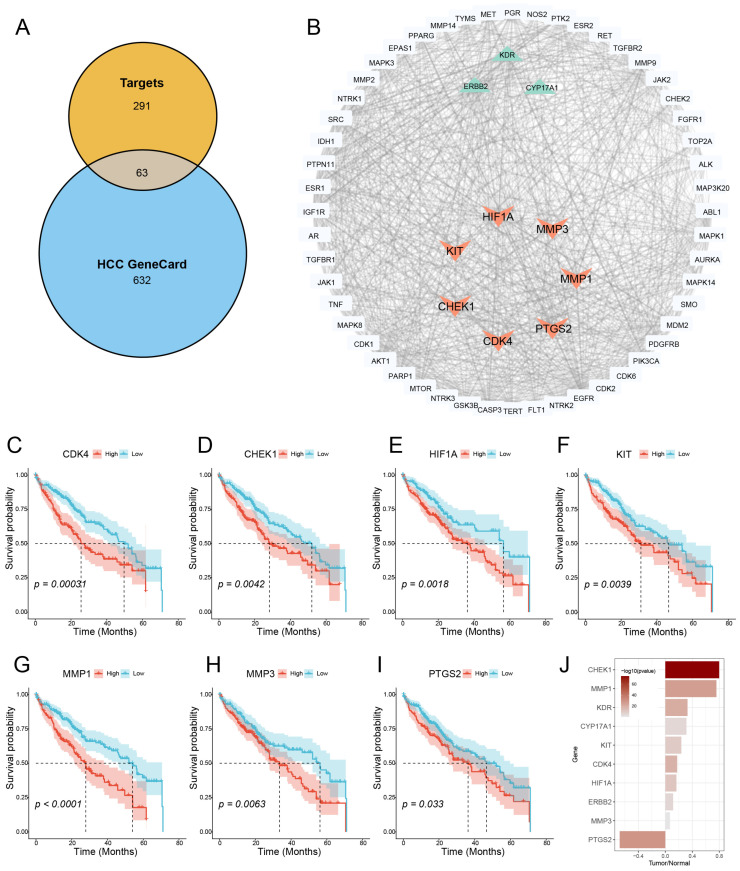
Screening of core therapeutic targets of *Polyporus umbellatus* against hepatocellular carcinoma and clinical prognostic analysis. (**A**) A Venn diagram illustrating the intersection between the predicted targets of *Polyporus umbellatus* active ingredients (354 targets) and hepatocellular carcinoma (HCC)-related genes (695 genes, GeneCards relevance score > 20), identifying 63 common targets. (**B**) A protein–protein interaction (PPI) network of the 63 intersection genes. Nodes are coded by color and shape based on their correlation with patient survival prognosis: green upward-facing triangles represent genes with a Hazard Ratio (HR) < 1, indicating protective prognostic factors; orange downward-facing triangles represent genes with an HR > 1, indicating poor prognostic factors; light gray circles represent genes with no statistically significant difference in survival. (**C**–**I**) Kaplan–Meier survival curves for selected key genes associated with poor prognosis (HR > 1) in HCC patients, including CDK4 (**C**), CHEK1 (**D**), HIF1A (**E**), KIT (**F**), MMP1 (**G**), MMP3 (**H**), and PTGS2 (**I**). Differences between high- and low-expression groups were assessed using the Log-rank test. (**J**) Differential expression analysis of key genes in HCC tumor tissues versus adjacent normal tissues from the TCGA-LIHC dataset, where color intensity represents the significance level (−log10(*p*-value)), and the bar direction indicates the fold change relative to normal tissue.

The elucidation of the molecular mechanisms by which *Polyporus umbellatus* exerts its anti-tumor effects requires a precise understanding of the interplay between the targets of bioactive compounds and disease-specific genetic alterations. By intersecting predicted targets of *Polyporus umbellatus* active ingredients with 695 high-confidence HCC-related genes, we identified 63 common targets (Figure 2A). This overlap represents the core therapeutic module, suggesting that the pharmacological efficacy of the herbal formulation is mediated through the modulation of these specific disease-associated proteins.

To further prioritize these targets based on their clinical relevance, we constructed a protein–protein interaction network that integrates survival data from the TCGA-LIHC cohort (Figure 2B). This network reveals a complex interactome where the nodes are differentiated by their prognostic significance. Notably, we observed a distinct cluster of genes associated with poor prognosis (HR > 1), represented by orange downward-pointing triangles, including *HIF1A*, *MMP1*, *MMP3*, *PTGS2*, *CDK4*, *KIT*, and *CHEK1*. Conversely, genes such as *KDR* and *ERBB2*, depicted as green upward-pointing triangles, were associated with a favorable prognosis (HR < 1), while gray nodes indicated genes with no statistically significant impact on survival. This topological stratification highlights that *Polyporus umbellatus* may function by simultaneously suppressing oncogenic drivers and potentially enhancing tumor-suppressive pathways.

We subsequently focused our analysis on the high-risk genes to validate their potential as therapeutic targets. Kaplan–Meier survival analysis demonstrated that high expression levels of *CDK4* (*p* = 0.00031), *CHEK1* (*p* = 0.0042), *HIF1A* (*p* = 0.0018), *KIT* (*p* = 0.0039), *MMP1* (*p* < 0.0001), *MMP3* (*p* = 0.0063), and *PTGS2* (*p* = 0.033) were significantly correlated with reduced overall survival in HCC patients (Figure 2C–I). These findings confirm that the upregulation of these genes contributes to adverse clinical outcomes, making them prime candidates for pharmacological inhibition. To corroborate these survival data with transcriptional dysregulation, we analyzed the differential expression of these genes in tumor versus normal tissues (Figure 2J). The analysis revealed that genes such as *CHEK1*, *MMP1*, and *CDK4* were significantly upregulated in tumor tissues, reinforcing their role in tumorigenesis. Among these candidates, *CHEK1* emerged as a particularly compelling target due to its dual characteristics: it is significantly overexpressed in HCC tissues and strongly associated with poor survival. Furthermore, molecular docking indicates that *CHEK1* is a primary binding target of polyporusterone E, a key bioactive constituent of *Polyporus umbellatus* (Supplementary Table S1). Consequently, *CHEK1* was selected as the focal point for further experimental validation to elucidate the specific molecular mechanism by which polyporusterone E arrests HCC progression.

### 2.3. Elucidation of Signal Transduction Cascades and Kinase-Modulated Biological Processes

**Figure 3:**
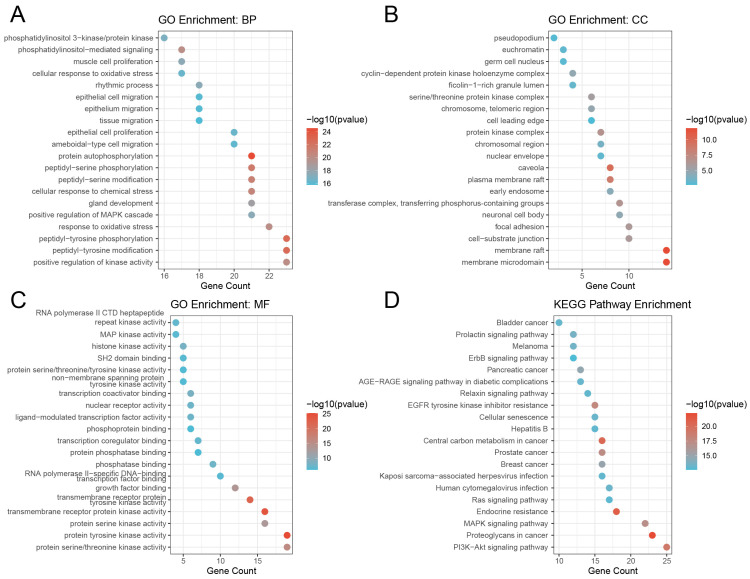
Gene Ontology (GO) functional annotation and Kyoto Encyclopedia of Genes and Genomes (KEGG) pathway enrichment analysis of the core intersection genes. (**A**) Enrichment analysis of Biological Processes (BPs). (**B**) Enrichment analysis of Cellular Components (CCs). (**C**) Enrichment analysis of Molecular Functions (MFs). (**D**) KEGG signaling pathway enrichment analysis. In the bubble plots, the color gradient indicates statistical significance (−log10(*p*-value)).

The functional characterization of the 63 core targets revealed a highly interconnected network of signaling pathways primarily governing cell proliferation and survival. The Gene Ontology enrichment analysis provided a granular view of the biological roles played by these targets. In the Biological Process category, the analysis highlighted a predominant involvement in phosphorylation-dependent signaling (Figure 3A). The most significantly enriched terms included the positive regulation of kinase activity, peptidyl-tyrosine modification, and peptidyl-tyrosine phosphorylation. Notably, the targets were also strongly enriched for positive regulation of the MAPK cascade and cellular response to oxidative stress, suggesting that *Polyporus umbellatus* may exert its therapeutic effects by modulating stress–response pathways and kinase-driven signal transduction.

Analysis of Cellular Components indicated that the protein products of these core targets are spatially concentrated in critical signaling hubs (Figure 3B). The most significant enrichment was observed in membrane microdomains and membrane rafts, which serve as organizing centers for the assembly of signaling molecules. Furthermore, significant enrichment in focal adhesions, cell–substrate junctions, and protein kinase complexes suggests that the active ingredients likely influence the structural integrity and communicative capacity of the tumor microenvironment, potentially affecting cancer cell migration and invasion.

Consistent with the BP and CC findings, the Molecular Function analysis confirmed that the core targets predominantly function as enzymatic regulators (Figure 3C). The top enriched terms were protein serine/threonine kinase activity, protein tyrosine kinase activity, and transmembrane receptor protein tyrosine kinase activity. This overwhelming enrichment in kinase activity underscores the potential of the identified compounds to act as multi-kinase inhibitors or modulators, directly interfering with the catalytic machinery that drives malignant progression.

To integrate these functional insights into a systemic context, we performed KEGG pathway enrichment analysis (Figure 3D). The results demonstrated that the core targets are intricately mapped to several canonical cancer-related pathways. The PI3K-Akt signaling pathway emerged as the most significantly enriched pathway with the highest gene count, indicating its central role in the drug’s mechanism of action. Additionally, the analysis revealed significant enrichment in the MAPK and Ras signaling pathways, as well as in Proteoglycans, in cancer. The convergence of these targets on the PI3K-Akt and MAPK axes suggests a dual-targeting mechanism in which the active ingredients of *Polyporus umbellatus* may simultaneously block two of the most critical survival pathways in hepatocellular carcinoma, thereby overcoming potential compensatory resistance mechanisms often observed with single-target therapies.

### 2.4. Validation of Ligand-Target Stability via Molecular Dynamics Simulations

To identify the most critical interactions for validation, we prioritized targets based on binding probability scores from the Swiss Target Prediction database. CHEK1 (O14757), KIT (P10721), PTGS2 (P35354), and HIF1A (Q16665) emerged as the top candidates, exhibiting the highest probability values among the screened proteins. Polyporusterone E was selected as the focal ligand for this analysis because it was predicted to target all four of these high-ranking proteins. We subsequently conducted 100 ns molecular dynamics simulations to rigorously evaluate the stability of polyporusterone E within the binding pockets of these targets. This dynamic approach extends beyond static docking scores by introducing time as a dimension, enabling assessment of thermodynamic stability and conformational evolution of ligand–protein complexes.

**Figure 4:**
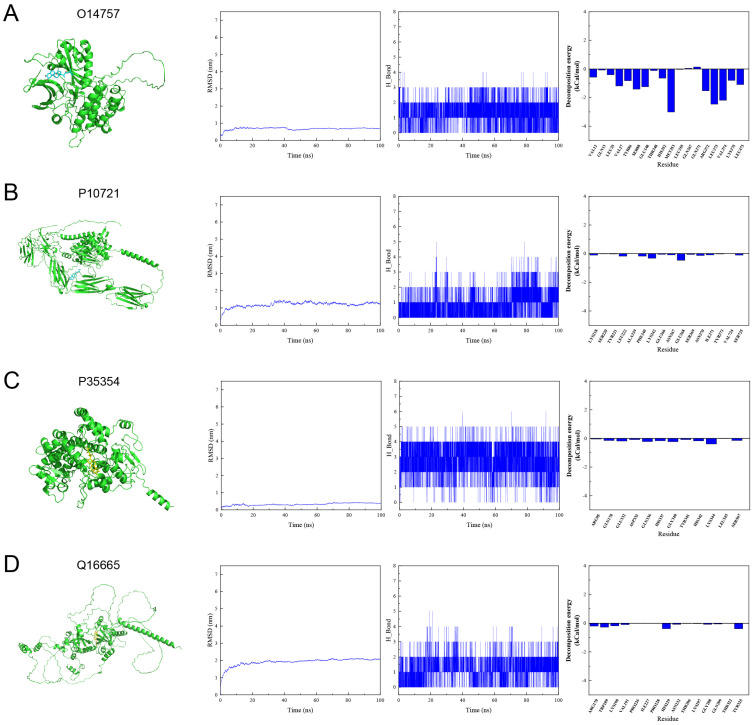
Molecular docking and molecular dynamics simulation validation of the binding stability between polyporusterone E and core targets. (**A**) The binding mode of the polyporusterone E-CHEK1 (O14757) complex (far left), RMSD evolution curve (middle left), hydrogen bond count over time (middle right), and decomposition of binding energy for key residues (far right). (**B**) Corresponding simulation data for the polyporusterone E-KIT (P10721) complex. (**C**) Corresponding simulation data for the polyporusterone E-PTGS2 (P35354) complex. (**D**) Corresponding simulation data for the polyporusterone E-HIF1A (Q16665) complex. The RMSD curves illustrate the structural stability of the complexes over the 100 ns simulation, while the binding energy decomposition plots display the energy contribution (kcal/mol) of the primary contributing amino acid residues.

The trajectory analysis revealed distinct dynamic behaviors among the different complexes. The CHEK1–polyporusterone E complex (Figure 4A) demonstrated a highly favorable binding profile. The Root Mean Square Deviation (RMSD) rapidly stabilized at approximately 0.6 nm within the first 10 ns and remained at this value throughout the simulation, indicating a robust, relaxed conformation. Similarly, the PTGS2 complex (Figure 4C) exhibited remarkable structural rigidity, with RMSD values consistently remaining below 0.4 nm. This low deviation suggests that polyporusterone E fits tightly within the PTGS2 binding pocket with minimal steric hindrance. In contrast, the trajectories for KIT (Figure 4B) and HIF1A (Figure 4D) displayed greater volatility. Specifically, the HIF1A complex showed a progressive increase in RMSD values up to 2.0 nm, implying significant conformational adjustments or a tendency toward dissociation, indicating a less stable interaction compared to CHEK1 and PTGS2.

While both CHEK1 and PTGS2 exhibited structural stability, a deeper analysis of the interaction energies highlights CHEK1’s superior binding affinity. The hydrogen bond analysis for CHEK1 (Figure 4A, third column) revealed a dense and persistent network of bonds, whereas the hydrogen bonding in the PTGS2 complex (Figure 4C, third column), although present, appeared less frequent and more transient. Furthermore, the per-residue energy decomposition analysis provided critical mechanistic insights. In the CHEK1 complex, several key residues, including Leucine (LEU354) and Methionine (MET353), contributed significantly to the binding free energy, with values exceeding −2 to −3 kcal/mol (Figure 4A, fourth column). Conversely, the energy decomposition for PTGS2 (Figure 4C, fourth column) showed that while multiple residues contributed to binding, the individual energetic contributions were generally weaker, mostly remaining above −1 kcal/mol. This distinction underscores that the interaction between polyporusterone E and CHEK1 is driven by stronger, specific molecular contacts.

To provide direct biophysical evidence for the computational prediction, we performed surface plasmon resonance (SPR) analysis using recombinant human CHEK1 protein. The SPR sensorgrams revealed concentration-dependent binding of polyporusterone E to CHEK1, with a kinetic dissociation constant (K_D) of 1.38 μM and a steady-state K_D of 3.75 μM (Supplementary Figure S1). These results experimentally confirm that polyporusterone E physically interacts with the CHEK1 protein, corroborating the molecular dynamics simulation predictions.

Collectively, these data suggest that while polyporusterone E exhibits a good structural fit with PTGS2, it forms the most energetically favorable and stable complex with CHEK1. The combination of stable RMSD, persistent hydrogen bonding, and high-affinity residue interactions identifies CHEK1 as the most probable primary biological target for polyporusterone E.

### 2.5. Oncogenic Functionality of CHEK1 in Driving Hepatocellular Carcinoma Proliferation and Metastasis

**Figure 5:**
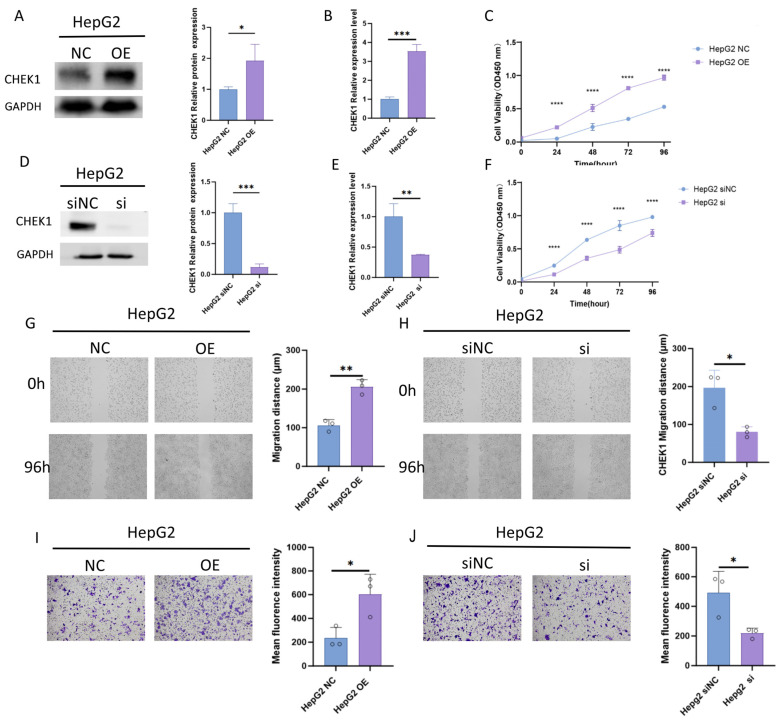
CHEK1 promotes the malignant behaviors of HepG2 hepatocellular carcinoma cells. (**A**) Western blot analysis verifying the transfection efficiency of CHEK1 overexpression (OE) in HepG2 cells compared to the negative control (NC). (**B**) Quantitative real-time PCR analysis of CHEK1 mRNA levels in HepG2 cells following overexpression. (**C**) CCK-8 assay illustrating the proliferation curves of HepG2 cells in the NC and OE groups over 96 h. (**D**) Western blot analysis confirming CHEK1 knockdown efficiency using siRNA in HepG2 cells. (**E**) Quantitative real-time PCR analysis of CHEK1 mRNA levels in HepG2 cells following siRNA-mediated knockdown. (**F**) CCK-8 assay showing the proliferation rates of HepG2 cells in the siNC and siCHEK1 groups. (**G**) Representative images and quantification of the wound healing assay assessing the migration ability of HepG2 cells upon CHEK1 overexpression. (**H**) Representative images and quantification of the wound healing assay assessing the migration ability of HepG2 cells upon CHEK1 knockdown. (**I**) Transwell invasion assay images and quantification of invaded HepG2 cells in the NC and OE groups. (**J**) Transwell invasion assay images and quantification of invaded HepG2 cells in the siNC and siCHEK1 groups. Data are presented as mean ± SD, n = 3 independent biological replicates (* *p* < 0.05, ** *p<* 0.01, *** *p* < 0.001, **** *p* < 0.0001).

**Figure 6:**
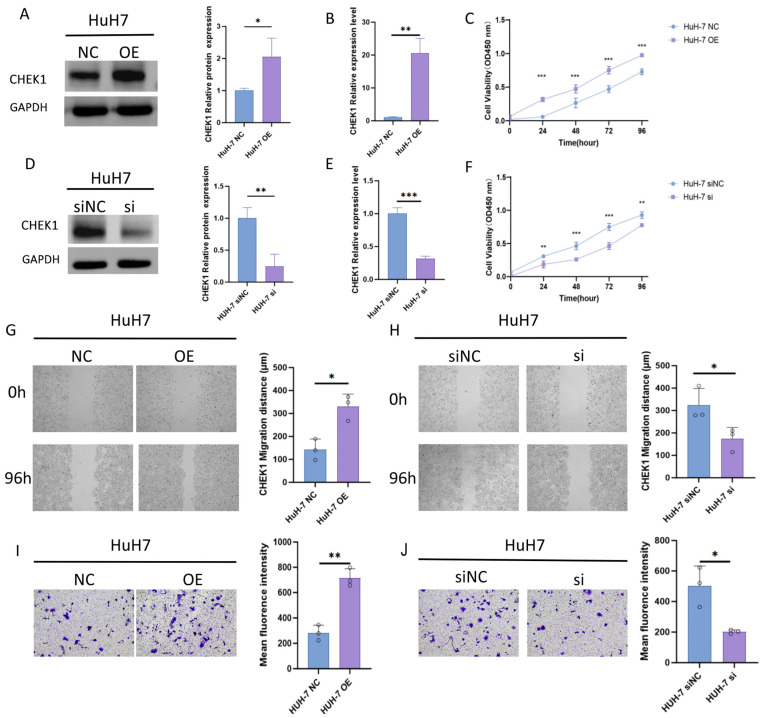
CHEK1 promotes the malignant behaviors of HuH-7 hepatocellular carcinoma cells. (**A**) Western blot analysis verifying the transfection efficiency of CHEK1 overexpression (OE) in HuH-7 cells compared to the negative control (NC). (**B**) Quantitative real-time PCR analysis of CHEK1 mRNA levels in HuH-7 cells following overexpression. (**C**) CCK-8 assay illustrating the proliferation curves of HuH-7 cells in the NC and OE groups over 96 h. (**D**) Western blot analysis confirming the knockdown efficiency of CHEK1 using siRNA in HuH-7 cells. (**E**) Quantitative real-time PCR analysis of CHEK1 mRNA levels in HuH-7 cells following siRNA-mediated knockdown. (**F**) CCK-8 assay showing the proliferation rates of HuH-7 cells in the siNC and siCHEK1 groups. (**G**) Representative images and quantification of the wound healing assay assessing the migration ability of HuH-7 cells upon CHEK1 overexpression. (**H**) Representative images and quantification of the wound healing assay assessing the migration ability of HuH-7 cells upon CHEK1 knockdown. (**I**) Transwell invasion assay images and quantification of invaded HuH-7 cells in the NC and OE groups. (**J**) Transwell invasion assay images and quantification of invaded HuH-7 cells in the siNC and siCHEK1 groups. Data are presented as mean ± SD, n = 3 independent biological replicates (* *p* < 0.05, ** *p* < 0.01, *** *p* < 0.001).

To delineate the biological significance of CHEK1 in the progression of hepatocellular carcinoma, we systematically modulated its expression in HepG2 and HuH-7 cell lines and assessed the consequent phenotypic alterations. The successful establishment of these experimental models was confirmed through rigorous molecular validation. In both HepG2 (Figure 5A,B,D,E) and HuH-7 (Figure 6A,B,D,E) cells, Western blot and quantitative real-time polymerase chain reaction analyses demonstrated that the overexpression vectors significantly elevated CHEK1 protein and mRNA levels, whereas the specific siRNA constructs effectively silenced endogenous CHEK1 expression compared with their respective negative controls.

Following this validation, we investigated the impact of CHEK1 on cellular proliferation dynamics. The CCK-8 assays revealed a direct correlation between CHEK1 levels and cell growth rates. Overexpression of CHEK1 in HepG2 (Figure 5C) and HuH-7 (Figure 6C) cells resulted in a marked increase in optical density values over a 96 h period, indicating an enhanced proliferative capacity. Conversely, CHEK1 depletion significantly suppressed the growth of both cell lines (Figure 5F and Figure 6F), suggesting that CHEK1 is indispensable for the sustained proliferation of these hepatoma cells.

Beyond proliferation, the metastatic potential of tumor cells, characterized by their ability to migrate and invade, is a critical determinant of patient prognosis. Our wound healing assays provided compelling evidence that CHEK1 modulation alters cellular motility. In the overexpression groups, HepG2 and HuH-7 cells exhibited accelerated wound closure rates compared with controls (Figure 5G and Figure 6G), implying an enhanced migratory phenotype. In sharp contrast, CHEK1 knockdown significantly impaired cell migration, resulting in wider residual gaps at the experimental endpoint (Figure 5H and Figure 6H). These findings were further corroborated by Transwell invasion assays, which offer a three-dimensional assessment of the cells’ ability to traverse the extracellular matrix. The results unequivocally showed that CHEK1 upregulation significantly increased the number of invaded cells (Figure 5I and Figure 6I), whereas its downregulation drastically reduced the invasive population (Figure 5J and Figure 6J).

### 2.6. Pharmacological Suppression of CHEK1 by Polyporus umbellatus and Polyporusterone E Compromises Hepatocellular Carcinoma Viability

Natural products have long served as a repository for identifying novel antineoplastic agents, and understanding their molecular targets is essential for their translation into clinical therapeutics. In this phase of the study, we sought to determine whether the observed oncogenic dependency of hepatocellular carcinoma cells on CHEK1 could be therapeutically exploited using *Polyporus umbellatus*, a traditional medicinal fungus, and its major bioactive constituent, polyporusterone E. We hypothesized that the anti-tumor efficacy of these agents is mediated, at least in part, through the downregulation of CHEK1 expression. To evaluate the dose-dependent effects of *Polyporus umbellatus* extract and polyporusterone E on HCC cell viability, multi-concentration CCK-8 assays were performed. As shown in Supplementary Figure S2, both compounds exhibited dose-dependent inhibitory effects on cell viability in HepG2 and HuH-7 cells. Polyporusterone E exhibited approximately four-fold greater potency (IC_50_ 40.91–45.60 μg/mL) against HepG2 and HuH-7 cells compared to the crude *Polyporus umbellatus* extract (IC_50_ 184.1–200.2 μg/mL), confirming that polyporusterone E is a key pharmacophore responsible for the anti-proliferative activity of the parent fungal extract.

**Figure 7:**
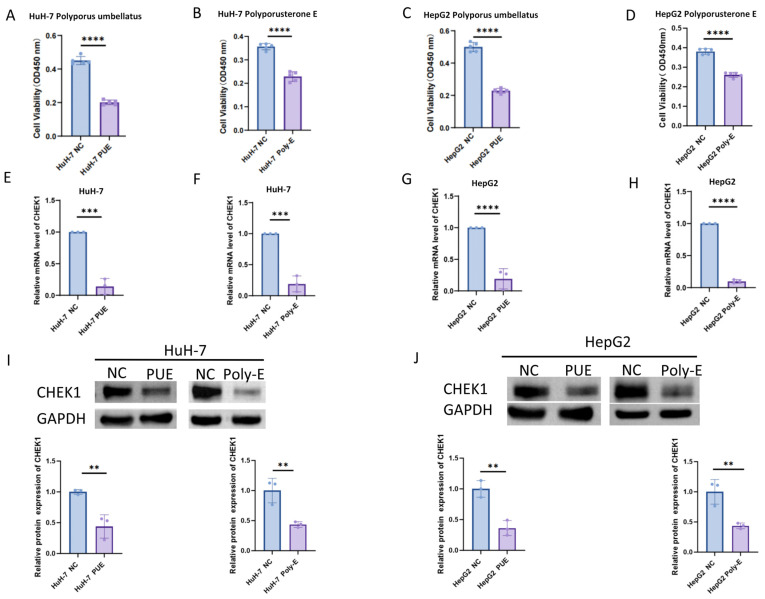
*Polyporus umbellatus* extract and its active constituent polyporusterone E attenuate hepatocellular carcinoma cell viability by suppressing CHEK1 expression. (**A**) The cell viability of HuH-7 cells treated with PUE (*Polyporus umbellatus* extract) assessed using a CCK-8 assay. (**B**) The cell viability of HuH-7 cells treated with Poly-E (polyporusterone E) assessed using a CCK-8 assay. (**C**) The cell viability of HepG2 cells treated with *Polyporus umbellatus* extract assessed using a CCK-8 assay. (**D**) The cell viability of HepG2 cells treated with polyporusterone E assessed using a CCK-8 assay. (**E**) The relative mRNA expression of CHEK1 in HuH-7 cells following treatment with *Polyporus umbellatus* extract. (**F**) The relative mRNA expression of CHEK1 in HuH-7 cells following treatment with polyporusterone E. (**G**) The relative mRNA expression of CHEK1 in HepG2 cells following treatment with *Polyporus umbellatus* extract. (**H**) The relative mRNA expression of CHEK1 in HepG2 cells following treatment with polyporusterone E. (**I**) Western blot analysis and quantification of CHEK1 protein levels in HuH-7 cells treated with PUE and Poly-E. (**J**) Western blot analysis and quantification of CHEK1 protein levels in HepG2 cells treated with PUE and Poly-E. Data are presented as mean ± SD, n = 3 independent biological replicates (** *p* < 0.01, *** *p* < 0.001, **** *p* < 0.0001).

Initial assessment of cellular viability using the CCK-8 assay revealed potent cytotoxic effects of both the crude extract and the purified monomer. Treatment with *Polyporus umbellatus* extract significantly reduced the optical density values in HuH-7 (Figure 7A) and HepG2 (Figure 7C) cells compared to the negative control groups, indicating a substantial inhibition of cell proliferation. Crucially, polyporusterone E recapitulated these effects, inducing a marked decrease in viability in both HuH-7 (Figure 7B) and HepG2 (Figure 7D) cell lines. These data are consistent with the dose–response analysis and support the contribution of polyporusterone E to the anti-proliferative activity observed for the parent fungal extract.

To elucidate the molecular mechanism driving this growth inhibition, we examined the transcriptional landscape of *CHEK1* following drug exposure. Quantitative real-time polymerase chain reaction analysis demonstrated a precipitous drop in *CHEK1* mRNA levels in cells treated with *Polyporus umbellatus*. Specifically, relative expression levels were significantly decreased in HuH-7 (Figure 7E) and HepG2 (Figure 7G) cells. Similarly, treatment with polyporusterone E resulted in a robust suppression of *CHEK1* transcripts in both cell lines (Figure 7F,H), suggesting that these agents act by interfering with the transcriptional activation or stability of *CHEK1* mRNA.

We further corroborated these findings at the translational level using Western blot analysis. The protein abundance of CHEK1 was visibly diminished following treatment with *Polyporus umbellatus* in HuH-7 and HepG2 cells (Figure 7I,J). Consistent with the mRNA data, polyporusterone E treatment also led to a profound reduction in CHEK1 protein levels in both HuH-7 and HepG2 cells (Figure 7I,J).

## 3. Discussion

The systematic elucidation of the pharmacological basis and molecular mechanisms of TCM is pivotal for its modernization and integration into precision oncology. In this study, we employed a comprehensive strategy that integrated network pharmacology, molecular dynamics simulations, and biological validation to elucidate the anti-HCC effects of *Polyporus umbellatus*. Our investigation revealed that the therapeutic efficacy of *Polyporus umbellatus* is not solely reliant on its well-documented polysaccharides but is significantly mediated by a cluster of bioactive ergostane-type triterpenoids. Specifically, our data suggest that polyporusterone E is a bioactive constituent of interest that may suppress HCC cell proliferation, potentially through the downregulation of CHEK1 expression, although the direct causal relationship remains to be established.

A critical finding of our study is the identification of 11 core bioactive ingredients, predominantly sterols such as ergosta-7,22-diene-3β-ol and polyporusterone E, which exhibit favorable pharmacokinetic profiles. Historically, research on *Polyporus umbellatus* has largely focused on its polysaccharide fraction, attributing its anti-tumor properties primarily to immunomodulatory and antioxidant activities [20]. While polysaccharides are undoubtedly important, our results highlight the previously underappreciated cytotoxic potential of small-molecule sterols in this fungal species. This aligns with recent evidence suggesting that ergosterol derivatives from medicinal fungi exhibit significant antiproliferative activity against various cancer cell lines [21,22]. By constructing a compound–target network, we observed that these sterols are computationally predicted to interact with multiple biological targets, suggesting a multi-target mode of action that warrants experimental confirmation. It is important to acknowledge that although polyporusterone E was computationally prioritized as a candidate compound associated with CHEK1, the overall biological outcome is likely influenced by multiple components in the extract, and that designating polyporusterone E as the primary effector requires further functional validation, such as rescue experiments. Other co-existing sterols may act as adjuvant agents, potentially enhancing the bioavailability of polyporusterone E or simultaneously targeting compensatory survival pathways. Such multi-component interplay is considered a characteristic feature of TCM pharmacology, though whether it confers meaningful advantages over single-target therapies in the context of HCC remains an open question that would require rigorous comparative studies to address.

A central aim of this work was to investigate CHEK1 as a candidate therapeutic target associated with the anti-HCC activity of *Polyporus umbellatus*. Our survival analysis of the TCGA-LIHC cohort confirmed that CHEK1 overexpression is strongly correlated with poor clinical prognosis, consistent with its established role in mediating the DNA damage response and cell cycle checkpoints [16,23]. Although synthetic CHEK1 inhibitors are currently under clinical investigation, to our knowledge, this study represents an early attempt to explore whether a natural ergosterol derivative from *Polyporus umbellatus* may modulate CHEK1 expression in HCC cells. Notably, among the 63 intersecting targets identified in this study, several genes beyond CHEK1 demonstrated significant associations with poor prognosis in HCC. Specifically, CDK4, a cyclin-dependent kinase essential for the G1/S cell cycle transition, has been identified as a promising therapeutic target in HCC, with its overexpression correlating with advanced tumor stage and unfavorable clinical outcomes [24,25]. HIF1A, a master regulator of the cellular response to hypoxia, plays a critical role in promoting angiogenesis, metabolic reprogramming, and metastasis within the hypoxic tumor microenvironment of HCC [26]. MMP1, a matrix metalloproteinase involved in extracellular matrix degradation, has been implicated in HCC invasion and metastatic dissemination [27,28]. Although our experimental validation focused on CHEK1, these additional targets may also contribute to the anti-HCC effects of *Polyporus umbellatus* and warrant systematic investigation in future studies. The concurrent modulation of multiple oncogenic targets by *Polyporus umbellatus* active ingredients aligns with the multi-target therapeutic paradigm characteristic of traditional medicine-derived compounds.

The molecular docking and molecular dynamics simulations provided a structural basis for this interaction, revealing that polyporusterone E forms a stable complex with CHEK1, characterized by low RMSD values and persistent hydrogen bonding. SPR analysis further provided direct biophysical evidence that polyporusterone E physically binds recombinant human CHEK1 protein. However, whether this binding alters CHEK1 kinase activity, substrate phosphorylation, or downstream cell-cycle checkpoint signaling remains to be determined. Therefore, polyporusterone E should be regarded at this stage as a potential CHEK1-binding modulator rather than a validated CHEK1 enzymatic inhibitor.

Furthermore, our pathway enrichment analysis revealed that the core targets of *Polyporus umbellatus* are significantly enriched in the PI3K-Akt and MAPK signaling pathways. These pathways are hyperactivated in the majority of HCC cases and are central regulators of cell survival, proliferation, and metastasis [29]. The convergence of the predicted targets on both the PI3K-Akt and MAPK axes suggests that *Polyporus umbellatus* active ingredients may influence multiple oncogenic pathways simultaneously. Whether such multi-pathway engagement reduces drug resistance compared with single-pathway therapies remains speculative at this stage and would need to be tested in appropriate preclinical resistance models. Our in vitro experiments demonstrated that *Polyporus umbellatus* extract and polyporusterone E can reduce CHEK1 expression and inhibit proliferation and migration in HepG2 and HuH-7 cells. These observations are consistent with, though not definitive proof of, the computationally predicted interaction between polyporusterone E and CHEK1. This validates the reliability of our network pharmacology-based approach and confirms that the anti-tumor mechanism involves disrupting kinase-driven signal transduction. Among the 11 bioactive components identified through ADME screening, polyporusterone E was prioritized for experimental validation based on a systematic computational ranking strategy. Among all 11 compounds, polyporusterone E was predicted to interact with the largest number of top-ranked target proteins (CHEK1, KIT, PTGS2, and HIF1A) based on SwissTargetPrediction probability scores. Furthermore, molecular dynamics simulations demonstrated that the polyporusterone E–CHEK1 complex exhibited the most thermodynamically stable binding profile, characterized by the lowest RMSD, the most persistent hydrogen bond network, and the highest per-residue binding energy contributions among all ligand–target combinations evaluated. This computational prioritization strategy, in which the top-ranked compound–target pair is selected for experimental validation, is consistent with the methodological framework widely adopted in network pharmacology studies [30,31]. Nevertheless, we acknowledge that other active components may also contribute to the observed anti-HCC effects through distinct or overlapping target profiles, and systematic functional characterization of all 11 compounds represents an important direction for future research.

Several important limitations of this study should be explicitly acknowledged. First, the causal link between polyporusterone E-mediated CHEK1 downregulation and the observed anti-tumor phenotype has not been established. Rescue experiments, in which CHEK1 is exogenously overexpressed during polyporusterone E treatment to determine whether the growth-inhibitory effects can be reversed, are essential to confirm that the drug’s efficacy is mechanistically dependent on CHEK1 suppression rather than being an epiphenomenon of general cytotoxicity. Second, although our SPR results (Supplementary Figure S1) provide direct biophysical evidence that polyporusterone E physically binds to CHEK1 protein (K_D = 1.38–3.75 μM), further functional validation using kinase activity assays is warranted to confirm that this binding translates to functional inhibition of CHEK1 enzymatic activity. Third, while multi-concentration CCK-8 assays with IC_50_ determination have been performed in the revised study (Supplementary Figure S2), no known CHEK1 inhibitor was included as a positive control, precluding a direct comparison of the inhibitory potency of polyporusterone E with established CHEK1-targeting agents such as Prexasertib. Fourth, given that CHEK1 is a canonical regulator of the DNA damage checkpoint, the absence of cell cycle distribution analysis and apoptosis detection (e.g., flow cytometry with Annexin V/PI staining) represents a notable gap in mechanistic characterization. Finally, all findings are based on in vitro experiments in two cell lines, and the pharmacokinetic behavior, metabolic stability, and therapeutic efficacy of polyporusterone E in vivo remain entirely unknown. Future work should prioritize the aforementioned mechanistic experiments alongside validation in orthotopic xenograft or patient-derived xenograft models.

## 4. Materials and Methods

### 4.1. Screening of Bioactive Ingredients and Target Prediction

The systematic identification of the pharmacological basis of *Polyporus umbellatus* was initiated by retrieving its chemical constituents from the Traditional Chinese Medicine Systems Pharmacology Database and Analysis Platform (TCMSP) [32,33,34]. To assess the therapeutic potential of the selected compounds, we conducted a rigorous pharmacokinetic evaluation based on Absorption, Distribution, Metabolism, and Excretion (ADME) parameters [12,35]. Two primary inclusion criteria were established to filter candidates: oral bioavailability (OB) greater than or equal to 30% and a drug-likeness (DL) index greater than or equal to 0.18 [12]. Molecules failing to meet these thresholds were excluded to ensure that subsequent analyses focused solely on constituents with high potential for clinical efficacy. Following the initial screening, the canonical SMILES strings for the identified bioactive compounds were obtained from the PubChem database to facilitate downstream target prediction [36,37].

### 4.2. Target Identification and Network Construction

The potential biological targets of the screened active ingredients were predicted using the SwissTargetPrediction web server, a tool that estimates the most probable protein targets of small molecules based on structural similarity and chemical properties [38,39]. We limited the species to “Homo sapiens” to ensure relevance to human liver cancer pathology. High-probability targets were collated and duplicate entries were removed to establish a unique gene set. To visualize the complex interactions between the bioactive ingredients and their corresponding targets, a compound–target (C-T) network was constructed. The network topology was visualized and analyzed using Cytoscape software (version 3.9.1) [40]. In this graphical representation, nodes were designated to represent the chemical compounds and protein targets, while edges symbolized the predicted interactions between them. This network-based approach enabled elucidation of the multi-component, multi-target regulatory mechanisms underlying the therapeutic action of *Polyporus umbellatus*.

### 4.3. Acquisition of Disease-Associated Targets and Intersection Analysis

To elucidate the genetic landscape of HCC and identify potential therapeutic targets, we systematically queried the GeneCards database, a comprehensive compendium of human genes [41,42]. The search strategy utilized the keywords “Hepatocellular Carcinoma” and “Liver Cancer” to retrieve disease-associated genes. To ensure the data’s relevance and reliability, a stringent relevance score threshold was applied; only gene targets with a relevance score greater than 20 were retained for subsequent analysis. This cutoff was adopted to retrieve genes with strong pathological relevance to HCC while maintaining a manageable gene set for downstream analysis, and it has been widely employed in previous network pharmacology studies investigating disease-related gene screening [43]. This filtration process was critical for excluding low-confidence associations and focusing on genes with established pathological significance in HCC. Subsequently, the potential targets of *Polyporus umbellatus* active ingredients identified in the preceding pharmacokinetic screening were mapped to the retrieved HCC-related genes. A Venn diagram was generated to visualize the intersection between the drug-target set and the disease-gene set. The genes located at this intersection were designated as potential core targets, representing the molecular interface through which the pharmacological activity of *Polyporus umbellatus* likely modulates the pathological network of HCC.

### 4.4. Construction of Protein–Protein Interaction Network and Prognostic Evaluation

The identified intersection genes were subjected to protein–protein interaction (PPI) analysis to decipher their functional connectivity. The interaction data were retrieved from the STRING database (Search Tool for the Retrieval of Interacting Genes/Proteins) [44], with the organism specified as *Homo sapiens*. High-confidence interaction data were imported into Cytoscape software (version 3.9.1) for network construction and topological visualization. To integrate clinical prognostic value into the network topology, we performed a survival analysis for each node using the Cancer Genome Atlas Liver Hepatocellular Carcinoma (TCGA-LIHC) dataset [45]. Univariate Cox regression analysis was conducted to calculate the Hazard Ratio (HR) and *p*-values for each gene. Based on these clinical metrics, the network nodes were visually stratified: genes with an HR less than 1 associated with a favorable prognosis were distinguished from those with an HR greater than 1 associated with a poor prognosis. Kaplan–Meier (KM) survival curves were plotted for key risk-associated genes to statistically validate the correlation between gene expression levels and overall survival (OS) in HCC patients. The statistical significance of the survival differences was assessed using the Log-rank test. Furthermore, the differential expression profiles of these key targets were analyzed by comparing tumor tissues with adjacent normal tissues using data from the TCGA-LIHC cohort, thereby validating their status as potential oncogenes or tumor suppressors within the context of *Polyporus umbellatus* intervention The expression ratio was calculated by dividing the expression level in LIHC tumor tissues by that in normal liver tissues using TCGA data. Statistical significance of differential expression in LIHC was evaluated using either Student’s *t*-test or a nonparametric test, as appropriate, to determine whether the gene was significantly upregulated.

### 4.5. Functional Enrichment and Pathway Analysis

To systematically decipher the biological functions and signaling mechanisms orchestrated by the identified core targets, we performed a comprehensive enrichment analysis using the intersection genes derived from the Venn analysis. The functional annotation was performed using the clusterProfiler package in the R statistical computing environment (version 4.2.0) [46]. We queried the Gene Ontology (GO) database to categorize the targets into three distinct ontologies: Biological Process (BP), Cellular Component (CC), and Molecular Function (MF). Simultaneously, pathway enrichment analysis was performed using the Kyoto Encyclopedia of Genes and Genomes (KEGG) database to identify high-level genomic functional information [47].

For the GO and KEGG analyses, the org.Hs.eg.db package served as the source for genome-wide annotation for *Homo sapiens*. To ensure statistical rigor, we applied a hypergeometric test to calculate the *p*-values, which were subsequently adjusted for multiple testing using the Benjamini–Hochberg method to control the False Discovery Rate (FDR). A strict cutoff criterion was established where the *p*- and q-values were required to be less than 0.05 to be considered statistically significant. The top enriched terms and pathways were visualized as bubble plots using the ggplot2 package. In these visualizations, the size of each bubble corresponds to the count of enriched genes associated with a specific term, while the color gradient represents the statistical significance, denoted by the negative logarithm of the *p*-value.

### 4.6. Target Prediction and Molecular Docking Protocol

To elucidate the potential binding modes between the bioactive compound polyporusterone E and its putative targets, we employed a systematic computational approach. Initially, the binding pockets of the candidate proteins were predicted using the DogSite3 server, which utilizes a grid-based method to identify potential active sites on the protein surface. Subsequently, molecular docking was performed using AutoDock Vina (version 1.1.2; Scripps Research, La Jolla, CA, USA) [48,49]. The receptor proteins were prepared by removing water molecules and co-crystallized ligands, followed by adding polar hydrogens and Kollman charges. The grid box was defined to encompass the predicted binding pockets, ensuring sufficient space for ligand conformational sampling. The docking results were evaluated using affinity scores, and the conformations with the lowest binding energy were selected as the initial coordinates for subsequent molecular dynamics simulations.

### 4.7. Molecular Dynamics Simulation Setup and Analysis

To evaluate the thermodynamic stability and dynamic behavior of the ligand–protein complexes under physiological conditions, molecular dynamics (MD) simulations were conducted using the GROMACS software package (version 2025.4) [50]. The protein topology was generated using the pdb2gmx module with the Amber99sb-ildn force field, while the ligand topology was parameterized to be compatible with the protein force field. The complex was immersed in a cubic box solvated with the TIP3P water model. To neutralize the net charge of the system, an appropriate number of sodium ions (Na^+^) were added, replacing solvent molecules where necessary.

Prior to the production run, the system underwent energy minimization using the steepest descent algorithm to eliminate steric clashes and relax the structure. Following minimization, the system was equilibrated in two phases. First, an NVT ensemble (constant number of particles, volume, and temperature) simulation was performed for 100 ps to stabilize the temperature at 300 K. This was followed by an NPT ensemble (constant number of particles, pressure, and temperature) simulation for 100 ps to equilibrate the system pressure at 1 bar. The temperature was regulated using the V-rescale thermostat, while the pressure was maintained using the Parrinello–Rahman barostat.

The production MD simulation was carried out for 100 ns with a time step of 2 fs, totaling 50,000,000 steps. The LINCS algorithm was applied to constrain bond lengths involving hydrogen atoms, thereby allowing a 2 fs integration step. Long-range electrostatic interactions were calculated using the Particle Mesh Ewald (PME) method. Upon completion, the trajectory data were analyzed using built-in GROMACS tools. We calculated the Root Mean Square Deviation (RMSD) to assess structural stability, the Root Mean Square Fluctuation (RMSF) to evaluate residue flexibility, and the radius of gyration to monitor protein compactness. Furthermore, the stability of the interaction was characterized by analyzing the number of hydrogen bonds formed between the ligand and the protein over time. Finally, the binding free energy and per-residue energy decomposition were computed using the Molecular Mechanics Generalized Born Surface Area (MM/GBSA) method to identify key amino acid residues contributing to the binding affinity.

### 4.8. Cell Culture and Transfection

The human hepatocellular carcinoma cell lines HepG2 and HuH-7 were obtained from the American Type Culture Collection and cultured in Dulbecco’s Modified Eagle Medium supplemented with 10% fetal bovine serum and 1% penicillin–streptomycin antibiotics. All cell lines were maintained in a humidified incubator at 37 °C with 5% carbon dioxide. To modulate the expression levels of Checkpoint Kinase 1, lentiviral vectors encoding small interfering RNA targeting CHEK1 (siCHEK1) and a CHEK1 overexpression plasmid were constructed. Cells were seeded into 6-well plates and infected with the respective lentiviruses or control vectors (siNC and OE-NC) when they reached approximately 60% confluence. The specific target sequences employed for siRNA-mediated knockdown were CHEK1 human-1 (5′-CAAGAUGUGUGGUACUUUATT-3′), CHEK1 human-2 (5′-GACACUUCCUGAAGAUUAATT-3′), and CHEK1 human-3 (5′-CAAAUUGGAUGCAGACAAATT-3′). Stable cell lines were selected using puromycin for subsequent experiments. The efficiency of knockdown and overexpression was rigorously validated at both transcriptional and translational levels.

### 4.9. Quantitative Real-Time Polymerase Chain Reaction

Total RNA was extracted from the cultured cells using a TRIzol-equivalent RNA extraction kit (CW0560S, CWBIO, Beijing, China) following the manufacturer’s standard protocol. The concentration and purity of the isolated RNA were assessed using a spectrophotometer. Subsequently, reverse transcription was performed to synthesize complementary DNA using a high-capacity cDNA reverse transcription kit. Quantitative real-time polymerase chain reaction was conducted using an SYBR Green Master Mix on a real-time PCR system. The relative expression levels of CHEK1 were calculated using the 2^-ΔΔCt method, with Glyceraldehyde-3-phosphate dehydrogenase (GAPDH) serving as the internal normalization control. The specific primer sequences synthesized for this study were as follows. For CHEK1, the forward primer was 5′-CAGACTTTGGCTTGGCAACA-3′, and the reverse primer was 5′-TCCAGCGAGCATTGCAGTAA-3′. For the internal control GAPDH, the forward primer was 5′-CTTCCTTCCTGGGCATGG-3′, and the reverse primer was 5′-GCCGCCAGACAGCACTGT-3′. All qRT-PCR experiments were performed with at least three independent biological replicates, each containing three technical replicates. Data are presented as mean ± SD.

### 4.10. Western Blot Analysis

Total protein was extracted from the treated cells using Radioimmunoprecipitation Assay lysis buffer supplemented with protease and phosphatase inhibitors. The protein concentration was determined using a Bicinchoninic Acid protein assay kit. Equal amounts of protein samples were separated by sodium dodecyl sulfate–polyacrylamide gel electrophoresis and transferred onto polyvinylidene fluoride membranes. The membranes were blocked with 5% non-fat milk and incubated overnight at 4 °C with primary antibodies against CHEK1 (Huabio, Cat. ET1609-71, 1:2000) and GAPDH (Huabio, Cat. EM1101, 1:2000). After washing, the membranes were incubated with horseradish peroxidase-conjugated secondary antibodies (Beyotime, Cat. A0208 and A0216, 1:20,000). Protein bands were visualized using an ECL ultra-sensitive chemiluminescence kit (Xinhai Gene, Cat. M2301) and detected with an Odyssey imaging system (LI-COR, USA). All Western blot experiments were independently repeated at least 3 times.

### 4.11. Cell Proliferation Assay

Cell proliferation capabilities were assessed using the Cell Counting Kit-8 (CCK-8) assay. Transfected HepG2 and HuH-7 cells were seeded into 96-well plates at a density of 2000 cells per well. At 0, 24, 48, 72, and 96 h, the CCK-8 reagent was added to each well, and the cells were incubated for an additional 2 h at 37 °C. The optical density at 450 nm was measured using a microplate reader to generate growth curves.

### 4.12. Wound Healing Migration Assay

To evaluate cell migration, cells were seeded into 6-well plates and cultured until they formed a confluent monolayer. A linear scratch was created in the cell monolayer using a sterile 200 μL pipette tip. The cells were then washed with phosphate-buffered saline to remove debris and cultured in serum-free medium to minimize the effect of cell proliferation on gap closure. Images of the wound area were captured at 0 h and at the endpoint (72 or 96 h) using an inverted microscope. The migration distance was quantified by analyzing changes in wound width using ImageJ software (version 1.54j; National Institutes of Health, Bethesda, MD, USA).

### 4.13. Transwell Invasion Assay

Cell invasion potential was examined using Transwell chambers equipped with 8 μm pore size polycarbonate membranes pre-coated with Matrigel. Cells were suspended in serum-free medium and seeded into the upper chamber, while medium containing 20% fetal bovine serum was added to the lower chamber to serve as a chemoattractant. After incubation for 24 h, non-invading cells on the upper surface of the membrane were removed with a cotton swab. The cells that had invaded the lower surface were fixed with methanol and stained with 0.1% crystal violet. The number of invading cells was counted in five randomly selected fields under a microscope.

### 4.14. Reagents and Drug Preparation

The standardized *Polyporus umbellatus* extract (Catalog No. 19034777) was purchased from Qingdao Ease Chemical Co., Ltd. (Qingdao, China). According to the manufacturer’s specification, the extract was prepared as a water extract of *Polyporus umbellatus*. The bioactive monomer polyporusterone E (Catalog No. 141360-92-1) was obtained from Hangzhou Dinghao Technology Co., Ltd. (Hangzhou, China). For in vitro experiments, stock solutions of both compounds were prepared by dissolving them in dimethyl sulfoxide (DMSO) and then sterilizing them by filtration through a 0.22 μm membrane. The stock solutions were stored at −20 °C until use. Polyporusterone E was prepared as a 10 mM stock solution, corresponding to approximately 4.61 mg/mL based on its molecular weight of 460.65 g/mol. The *Polyporus umbellatus* extract was prepared as a 500 mg/mL stock solution. Working solutions were freshly prepared by diluting the stocks into complete culture medium. For standard treatment experiments, 1 μL of the 10 mM polyporusterone E stock was added per 1 mL of culture medium to obtain a final concentration of 50 μM, corresponding to approximately 23.0 μg/mL. For the extract treatment, 1 μL of the 500 mg/mL stock was added per 1 mL of culture medium to obtain a final concentration of 500 μg/mL. Specifically, the *Polyporus umbellatus* extract was used at a final concentration of 500 μg/mL for Western blotting, quantitative real-time PCR, and CCK-8 assays. Polyporusterone E was administered at a final concentration of 50 μM. In all experimental groups, the final concentration of DMSO was maintained below 0.1% (*v*/*v*) to minimize solvent-induced cytotoxicity.

### 4.15. Pharmacological Treatment and Cell Viability Analysis

To evaluate the cytotoxic effects of the pharmacological agents, HepG2 and HuH-7 cells were seeded into 96-well plates at an optimized density of 3000 cells per well and allowed to adhere overnight. The culture medium was then replaced with fresh medium containing either the *Polyporus umbellatus* extract or polyporusterone E at predetermined concentrations. Cells treated with medium containing an equivalent volume of DMSO served as the negative control (NC). Following a 48 h incubation period, cell viability was quantified using the CCK-8 assay. The absorbance was measured at 450 nm using a microplate reader, and the optical density (OD) values were recorded to calculate the relative cell viability compared to the control group. To investigate the molecular mechanism underlying the drug-induced effects, cells were cultured in 6-well plates and treated with the respective compounds for 48 h.

For the dose–response experiments, HepG2 and HuH-7 cells were seeded into 96-well plates at a density of 3000 cells per well and allowed to adhere overnight. The culture medium was then replaced with fresh medium containing *Polyporus umbellatus* extract (0–320 μg/mL) or polyporusterone E (0–50 μg/mL) at the indicated concentrations. Cells treated with medium containing an equivalent volume of DMSO (<0.1%, *v*/*v*) served as the negative control. After 48 h of incubation, cell viability was quantified using the CCK-8 assay. The absorbance at 450 nm was measured, and IC_50_ values were calculated by fitting the dose–response data to a four-parameter logistic regression model using GraphPad Prism software (version 8.3.0; GraphPad Software, Boston, MA, USA).

### 4.16. Surface Plasmon Resonance (SPR) Analysis

SPR experiments were performed on a Biacore SPR system (Cytiva, Uppsala, Sweden) at 25 °C. Recombinant human CHEK1 protein was immobilized on a CM5 sensor chip (Cytiva) via standard amine coupling chemistry. Briefly, the chip surface was activated with a 1:1 mixture of 0.4 M EDC (1-ethyl-3-(3-dimethylaminopropyl)carbodiimide) and 0.1 M NHS (N-hydroxysuccinimide) at a flow rate of 10 μL/min for 420 s. CHEK1 protein was diluted to 50 μg/mL in 10 mM sodium acetate buffer (pH 4.0) and injected over the activated surface at 10 μL/min for 420 s. The remaining active ester groups were blocked with 1 M ethanolamine-HCl (pH 8.5) at 10 μL/min for 420 s. A reference channel was prepared identically without protein coupling for background subtraction. The running buffer was 1× PBS-P+ (pH 7.4). For kinetic analysis, polyporusterone E was serially diluted in running buffer supplemented with 5% (*v*/*v*) DMSO to concentrations ranging from 12.5 to 12,800 nM and injected at a flow rate of 30 μL/min with an association time of 100 s and a dissociation time of 120 s. Chip regeneration was performed with 10 mM glycine-HCl (pH 2.0) for 5 min between cycles. Kinetic parameters (k_a_, k_d) were determined by global fitting to a 1:1 Langmuir binding model, and the steady-state K_D was obtained by fitting the equilibrium response versus concentration data using Biacore evaluation software.

### 4.17. Instruments, Materials, and Reagents

The key instruments used in this study included a CO_2_ incubator (Thermo Fisher Scientific, Waltham, MA, USA), biosafety cabinet (Thermo Fisher Scientific), inverted microscope (Olympus, Tokyo, Japan), real-time quantitative PCR system (ABI 7500, Thermo Fisher Scientific), microplate reader (Molecular Devices, San Jose, CA, USA), electrophoresis and Western blot transfer apparatus (Bio-Rad, Hercules, CA, USA), NanoDrop 2000 spectrophotometer (Thermo Fisher Scientific), and low-temperature centrifuge (Thermo Fisher Scientific). A complete list of instruments and equipment is provided in Supplementary Table S2. Reagents and consumables, including antibodies, assay kits, and cell culture supplies, were obtained from commercial sources, as detailed in Supplementary Table S3.

### 4.18. Statistical Analysis

All quantitative data are presented as mean ± standard deviation (SD) from at least three independent experiments. Statistical comparisons between two groups were performed using Student’s *t*-test, while comparisons among multiple groups were analyzed using one-way analysis of variance (ANOVA) followed by Tukey’s post hoc test. All statistical analyses were conducted using GraphPad Prism software. A *p*-value of less than 0.05 was considered statistically significant. Significance levels are denoted as follows: * *p* < 0.05, ** *p* < 0.01, *** *p* < 0.001, and **** *p* < 0.0001.

## 5. Conclusions

This study employed an integrative approach combining network pharmacology, molecular dynamics simulations, and in vitro experiments to investigate the anti-hepatocellular carcinoma potential of *Polyporus umbellatus*. Through ADME-based screening, 11 bioactive ergostane-type sterols were identified, and intersection analysis with HCC-related genes yielded 63 candidate targets. Clinical data from the TCGA-LIHC cohort indicate that CHEK1 overexpression is associated with an unfavorable prognosis. Molecular dynamics simulations suggested that polyporusterone E may form a relatively stable complex with CHEK1. Functional experiments demonstrated that CHEK1 modulation affects the proliferative and metastatic behaviors of HepG2 and HuH-7 cells, and that treatment with *Polyporus umbellatus* extract or polyporusterone E reduced CHEK1 expression at both mRNA and protein levels while suppressing cell viability. These findings provide a preliminary framework suggesting that Polyporusterone E may modulate CHEK1 expression and CHEK1-associated signaling in HCC cells. The computational prediction of a polyporusterone E–CHEK1 interaction was supported by SPR analysis. However, definitive mechanistic conclusions await further validation, including CHEK1 kinase activity assays, functional rescue experiments, cell cycle and apoptosis profiling, and in vivo efficacy studies.

## Data Availability

The original contributions presented in this study are included in the article/Supplementary Material. Further inquiries can be directed to the corresponding authors.

## Supplementary Materials

The following supporting information can be downloaded at: https://www.mdpi.com/article/10.3390/ijms27135694/s1.

## Abbreviations

The following abbreviations are used in this manuscript:

ADME	Absorption, Distribution, Metabolism, and Excretion
ALD	Alcohol-Related Liver Disease
ATP	Adenosine Triphosphate
BP	Biological Process
BSA	Bovine Serum Albumin
CC	Cellular Component
CCK-8	Cell Counting Kit-8
cDNA	Complementary Deoxyribonucleic Acid
CHEK1	Checkpoint Kinase 1
Cox	Cox Proportional Hazards Regression
C-T	Compound–Target
DL	Drug-Likeness
DMSO	Dimethyl Sulfoxide
DNA	Deoxyribonucleic Acid
FDR	False Discovery Rate
GAPDH	Glyceraldehyde-3-Phosphate Dehydrogenase
GBSA	Generalized Born Surface Area
GO	Gene Ontology
HCC	Hepatocellular Carcinoma
HIF1A	Hypoxia-Inducible Factor 1 Subunit Alpha
HR	Hazard Ratio
KEGG	Kyoto Encyclopedia of Genes and Genomes
KIT	KIT Proto-Oncogene, Receptor Tyrosine Kinase
KM	Kaplan–Meier
LINCS	Linear Constraint Solver
MAPK	Mitogen-Activated Protein Kinase
MASLD	Metabolic Dysfunction-Associated Steatotic Liver Disease
MD	Molecular Dynamics
MF	Molecular Function
MM/GBSA	Molecular Mechanics Generalized Born Surface Area
MMP	Matrix Metalloproteinase
mRNA	Messenger Ribonucleic Acid
NC	Negative Control
NPT	Constant Number of Particles, Pressure, and Temperature
NVT	Constant Number of Particles, Volume, and Temperature
OB	Oral Bioavailability
OE	Overexpression
OS	Overall Survival
PCR	Polymerase Chain Reaction
PI3K	Phosphoinositide 3-Kinase
Poly-E	Polyporusterone E
PME	Particle Mesh Ewald
PPI	Protein–Protein Interaction
PTGS2	Prostaglandin–Endoperoxide Synthase 2
PUE	*Polyporus umbellatus* Extract
qRT-PCR	Quantitative Real-Time Polymerase Chain Reaction
Ras	Rat Sarcoma Viral Oncogene Homolog
RNA	Ribonucleic Acid
RMSD	Root Mean Square Deviation
RMSF	Root Mean Square Fluctuation
siRNA	Small interfering Ribonucleic Acid
STRING	Search Tool for the Retrieval of Interacting Genes/Proteins
TCGA-LIHC	The Cancer Genome Atlas Liver Hepatocellular Carcinoma
TCM	Traditional Chinese Medicine
TCMSP	Traditional Chinese Medicine Systems Pharmacology Database and Analysis Platform
TIP3P	Transferable Intermolecular Potential 3-Point Water Model
TRIzol	Guanidinium Thiocyanate–Phenol–Chloroform Reagent
