# Supplementary material for: Polyporusterone E, a Key Component of Polyporus umbellatus, as a Potential Regulator of CHEK 1 in Liver Cancer: Integrated Network Pharmacology, Bioinformatics, and Experimental Validation
Source: Int J Mol Sci. 2026 Jun 24;27(13):5694. doi: 10.3390/ijms27135694 (PMC13362459; doi:10.3390/ijms27135694)
Supplement: Supplementary file 1 [file ijms-27-05694-s001.zip › ijms-4341283-supplementary.pdf]

Supplementary Table S1. Molecular Docking and MM/GBSA Binding Free Energy Results of Polyporusterone E with Four Prioritized Target Proteins

Target Protein	UniProt ID	Vina Score (kcal/mol)	MM/GBSA ΔG (kcal/mol)	Binding Pocket Center (x, y, z)
CHEK1	O14757	−7.7	−51.70 ± 3.78	(−4.9, 8.5, −1.6)
KIT	P10721	−8.4	−35.33 ± 3.58	(−16.5, −14.5, −7.9)
PTGS2	P35354	−7.7	−36.51 ± 2.96	(1.1, 9.8, −24.3)
HIF1A	Q16665	−8.1	−41.77 ± 3.95	(−23.9, 14.1, 8.1)

Supplementary Table S2. List of Instruments and Equipment Used in This Study

No.	Instrument	Manufacturer
1.	CO_2 cell culture incubator	Thermo Fisher Scientific (Waltham, MA, USA)
2.	Biosafety cabinet	Thermo Fisher Scientific (Waltham, MA, USA)
3.	Inverted optical microscope	Olympus (Tokyo, Japan)
4.	Real-time quantitative PCR system (ABI 7500)	Thermo Fisher Scientific (Waltham, MA, USA)
5.	PCR amplification instrument	Bio-Rad (Hercules, CA, USA)
6.	NanoDrop 2000 spectrophotometer	Thermo Fisher Scientific (Waltham, MA, USA)
7.	Microplate reader	Molecular Devices (San Jose, CA, USA)
8.	Electrophoresis tank	Bio-Rad (Hercules, CA, USA)
9.	Western blot transfer tank	Bio-Rad (Hercules, CA, USA)
10.	Electrophoretic imaging analysis system	LI-COR (Lincoln, NE, USA)
11.	Low-temperature centrifuge	Thermo Fisher Scientific (Waltham, MA, USA)
12.	High-speed centrifuge	Eppendorf (Hamburg, Germany)
13.	Analytical balance	Sartorius (Göttingen, Germany)
14.	Pipettes	Eppendorf (Hamburg, Germany)
15.	Cell counter	Counting Star
16.	Constant-temperature water bath	Haier (Qingdao, China)
17.	Metal bath	SciLogex (Rocky Hill, CT, USA)
18.	−20 °C refrigerator	Haier (Qingdao, China)
19.	−80 °C ultra-low temperature freezer	Thermo Fisher Scientific (Waltham, MA, USA)
20.	Ultrapure water system	Thermo Fisher Scientific (Waltham, MA, USA)
21.	SPR system	Cytiva (Uppsala, Sweden)

Supplementary Table S3. List of Key Materials and Reagents Used in This Study

No.	Reagent/Material	Catalog No.	Manufacturer
1	Polyporus umbellatus standardized extract	19034777	Guidechem (Hangzhou, China)
2	Polyporusterone E (purity >98%, HPLC)	141360-92-1	Hem960 (Qingdao, China)

3	DMEM high-glucose medium	C11965500BT	Gibco
4	Fetal bovine serum (FBS)	S711-001S	LONSERA
5	Penicillin-streptomycin solution (100×)	15140-122	Gibco
6	Trypsin-EDTA (0.25%, with phenol red)	C25200056	Gibco
7	Dimethyl sulfoxide (DMSO)	D2650	Sigma-Aldrich
8	TRIzol-equivalent RNA extraction kit	CW0560S	CWBIO (Beijing, China)
9	Reverse transcription kit	CW2569	CWBIO (Beijing, China)
10	SYBR Green qPCR Master Mix	GK10002	GLPBIO
11	CCK-8 cell proliferation assay kit	GK10001	GLPBIO
12	BCA protein quantification kit	ADS001DL1	Edison (Shanghai, China)
13	RIPA lysis buffer (strong)	PS0013	Leagene (Beijing, China)
14	PMSF protease inhibitor	ST505	Beyotime (Shanghai, China)
15	Omni-Easy™ one-step PAGE gel kit (10%)	ADS008DY	Edison (Shanghai, China)
16	Anti-CHEK1 primary antibody	ET1609-71	Huabio (Hangzhou, China)
17	Anti-GAPDH primary antibody	EM1101	Huabio (Hangzhou, China)
18	HRP-conjugated anti-rabbit secondary antibody	A0208	Beyotime (Shanghai, China)
19	HRP-conjugated anti-mouse secondary antibody	A0216	Beyotime (Shanghai, China)
20	ECL ultra-sensitive chemiluminescence kit	M2301	Xinhai Gene
21	Non-fat dry milk (for blocking)	1172GR500	BioFroxx
22	Protein-free rapid blocking solution	ADS002ZF	Edison (Shanghai, China)
23	TBS buffer	BL602A	BioSharp (Hefei, China)
24	PBS buffer powder	BL601A	BioSharp (Hefei, China)
25	Glycine	1275GR500	BioFroxx
26	SDS	3250GR500	BioFroxx
27	Tris-base	1115KG001	BioSharp (Hefei, China)
28	β-Mercaptoethanol	M828395	Macklin (Shanghai, China)
29	Crystal violet staining solution	BS941	BioSharp (Hefei, China)
30	4% paraformaldehyde fixative	BL539A	BioSharp (Hefei, China)
31	Transwell inserts (8.0 μm, with 24-well plate)	3422	Corning
32	96-well cell culture plates	3599	Corning
33	24-well cell culture plates	3524	Corning
34	0.22 μm syringe filter	4612	PALL
35	15 mL centrifuge tubes	430790	Corning
36	100 mm culture dishes	430167	Corning
37	0.2 mL 96-well PCR plates	MP-96-HS-0200	BioSharp (Hefei, China)
38	qPCR sealing film	UC-500	Axygen
39	WB transfer sponge pads	OT103	Yamei
40	Puromycin	53-79-2	Solarbio

41	Endotoxin-free midi plasmid prep kit	CW2105S	CWBIO (Beijing, China)
42	DEPC-treated water	BL510B	BioSharp (Hefei, China)
43	Antibody stripping solution	P0025L	Beyotime (Shanghai, China)
44	Cell cryopreservation solution (non-programmed)	BL203B	BioSharp (Hefei, China)
45	CM5 sensor chip	BR100399	Cytiva (Uppsala, Sweden)
46	Recombinant human CHEK1 protein	HY-P75672	MCE

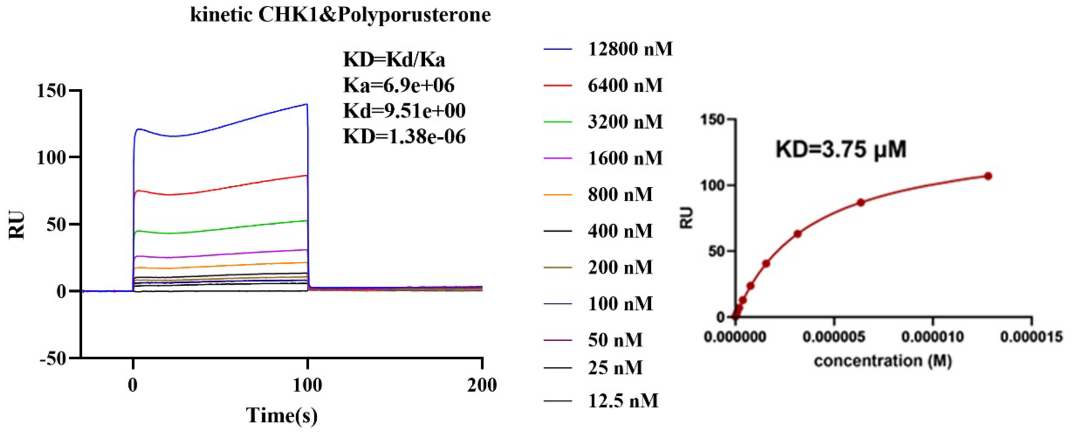

Supplementary Figure S1. Surface plasmon resonance (SPR) analysis of the direct binding interaction between Polyporusterone E and recombinant human CHEK1 protein. (Left panel) SPR sensorgrams showing the real-time binding responses (response units, RU) of Polyporusterone E at serial concentrations (12.5–12,800 nM) injected over a CHEK1-immobilized sensor chip surface. The association phase (0–100 s) and dissociation phase (100–200 s) are shown. The kinetic parameters were determined by global fitting to a 1:1 binding model, yielding an association rate constant (k_a) of 6.9×10^6 $M^{-1}s^{-1}$ and a dissociation rate constant (k_d) of 9.51 s^{-1}. (Right panel) Steady-state binding analysis plotting the equilibrium response (RU) against Polyporusterone E concentration (M). The data were fitted to a steady-state affinity model, yielding an equilibrium dissociation constant (K_D) of 3.75 μM. The kinetic K_D calculated from k_d/k_a was 1.38 μM.

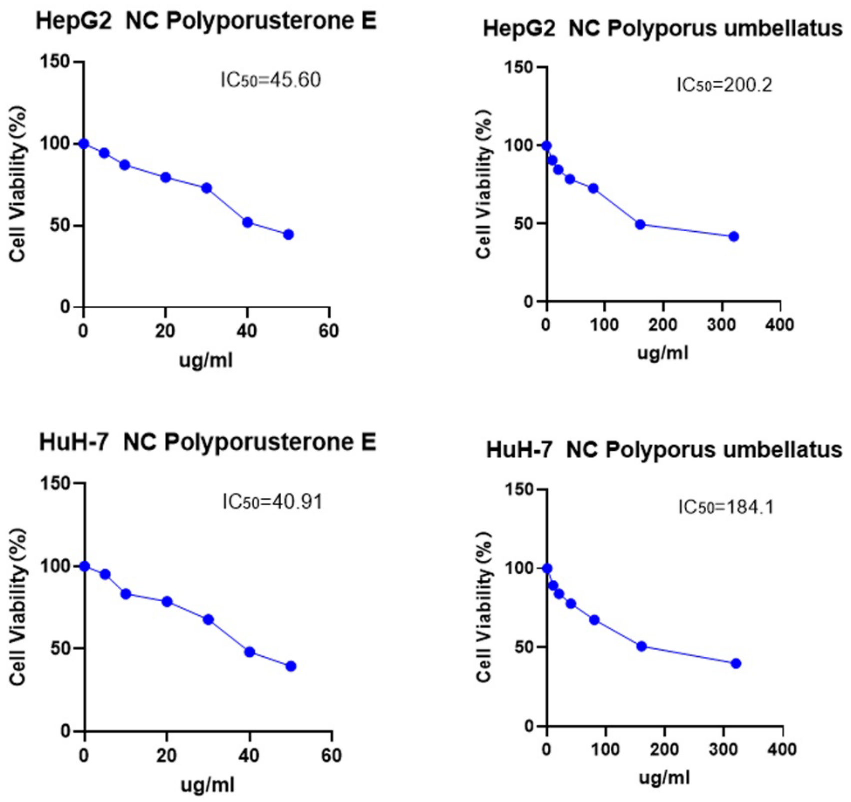

Supplementary Figure S2. **Dose-response curves for the cytotoxic effects of Polyporus umbellatus extract and Polyporusterone E on HCC cell lines.** Cell viability was assessed using the CCK-8 assay after 48 h of treatment. Dose-response curve of Polyporusterone E in HepG2 cells (IC_{50} = 45.60 μg/mL). Dose-response curve of *Polyporus umbellatus* extract in HepG2 cells (IC_{50} = 200.2 μg/mL). Dose-response curve of Polyporusterone E in HuH-7 cells (IC_{50} = 40.91 μg/mL). Dose-response curve of *Polyporus umbellatus* extract in HuH-7 cells (IC_{50} = 184.1 μg/mL). Data points represent mean values from replicate wells. IC_{50} values were calculated by nonlinear regression using a four-parameter logistic model. Both compounds exhibited dose-dependent inhibitory effects on cell viability in both HCC cell lines, with Polyporusterone E demonstrating approximately 4-fold greater potency than the crude extract.
